# Editorial: Lung monitoring in respiratory failure

**DOI:** 10.3389/fmed.2023.1155898

**Published:** 2023-02-28

**Authors:** Emanuele Rezoagli, Lu Chen, Giacomo Bellani

**Affiliations:** ^1^School of Medicine and Surgery, University of Milano-Bicocca, Monza, Italy; ^2^Department of Emergency and Intensive Care, Terapia intensiva e Semintensiva adulti e pediatrica, Fondazione Istituto di Ricovero e Cura a Carattere Scientifico (IRCCS) San Gerardo dei Tintori, Monza, Italy; ^3^Keenan Research Centre, St Michael's Hospital, Unity Health Toronto, Toronto, ON, Canada; ^4^Interdepartmental Division of Critical Care Medicine, University of Toronto, Toronto, ON, Canada

**Keywords:** prone position, esophageal pressure (Pes), surface electromyography (EMG), spontaneous breathing trial (SBT), mechanical ventilation, tracheal extubation, non-invasive ventilation (NIV)

Monitoring lung function and lung injury severity in patients with respiratory failure (1) is a crucial issue in critical care to deepen the understanding of patient respiratory mechanics, optimize the management of controlled and assisted mechanical ventilation and non-invasive respiratory support, and result in better clinical outcomes potentially.

Over the years, the development of lung monitoring technology has increased the tools available to improve the diagnostics of respiratory failure and the consequent mechanisms of impairment of the respiratory system function. Of note, during the recent COVID-19 pandemic, the need to better explore lung monitoring in the presence of respiratory failure increased (2).

In this Editorial, we highlight the key messages of the articles published in the Research Topic “*Lung monitoring in respiratory failure*” of Frontiers in Medicine, putting them in the context of the current literature.

## Lung monitoring during controlled mechanical ventilation

The beneficial role of prone positioning (PP) in COVID-19 patients on oxygenation was reported by Camporota et al.. This response was similar to the one observed in ARDS patients (3). In ARDS patients, Aguirre-Bermeo reported that end-expiratory lung volume (EELV) significantly increased after early prone positioning as compared to the supine position (SP), leading to reduced dynamic strain (4). In this issue of the journal, Dilken et al. explored the role of PP on lung resting volume by using the nitrogen washin/washout technique during a 16 h cycle. The authors observed that in severe COVID-19 ARDS, EELV steadily increased over time after PP showed the highest level at 16 h.

Meanwhile, dynamic strain gradually decreased, and oxygenation increased over time. Improvements in strain and oxygenation at the end of PP—and when patients were placed back to SP—were strongly associated with increased lung resting volume. Interestingly, after stratifying patients as responders and non-responders by assessing the increase or decrease of respiratory system compliance after PP, the authors reported that responders had a lower driving pressure over time and a higher Vt than non-responders. Furthermore, EELV/predicted body weight was higher and more elevated over time in responders, and so was oxygenation, while the ventilatory ratio decreased over time (Dilken et al.).

PP exploits gravity's effects, allowing a decrease in lung heterogeneity in the presence of lung injury. The use of gravity was extensively studied during the COVID pandemic. Maneuvers such as chest wall compressions unveiled the company of hidden over distension by analyzing lung volumes (5, 6), suggesting the need to optimize mechanical ventilation by reducing tidal volume or PEEP (7–9).

Current and future fields of investigation about preventing ventilator-induced lung injury include assessing the role and the distribution of controlled mechanical ventilation on the lung and the diaphragm by using non-invasive real-time dynamic monitoring tools such as ultrasound (10) and electrical impedance tomography (11).

## Lung monitoring during assisted mechanical ventilation and non-invasive respiratory support

The assessment of the inspiratory effort in patients undergoing non-invasive respiratory support or mechanical ventilation has a broad interest for multiple reasons, such as the prediction of the failure of non-invasive ventilation (12), the optimization of the patient-ventilator interaction (13) and the prevention of patient-self inflicted lung injury (14, 15).

A topical and timely field of research in lung monitoring is evaluating the inspiratory effort in patients undergoing weaning from mechanical ventilation. In this issue of the journal Pozzi et al. explored the role of accessory and expiratory muscles activation during a spontaneous breathing trial (SBT) by stratifying patients who failed or succeeded in ventilatory weaning. The authors evaluated the electrical activity (EA) of the diaphragm, accessory, and expiratory muscles using non-invasive surface electromyography (sEMG) in critically ill mechanically ventilated patients. The authors observed that the EA of respiratory muscles increased during SBT, regardless of SBT outcome. However, patients who failed the SBT showed a higher increase in the EA of all the inspiratory muscles than those who succeeded in the SBT. Furthermore, the recruitment of expiratory muscles—assessed by sEMG—was associated with SBT failure (Pozzi et al.). A non-invasive technique of evaluating the EA of inspiratory and expiratory muscles by sEMG proved feasible during SBT. It confirmed similar findings using more invasive procedures such as invasive electromyography (16) and the esophageal NAVA catheter (17).

The investigation of the inspiratory effort as a determinant of barotrauma was presented in an elegant paper by Tonelli et al. in this Research Topic. In COVID-19 patients with acute respiratory failure undergoing non-invasive respiratory support, not the esophageal pressure but the dynamic transpulmonary pressure swings were associated with the onset of air leak (AL). Furthermore, positive end-expiratory pressure (PEEP) and pressure support levels were associated with the AL onset (Tonelli et al.). This work confirms the role of elevated positive pressure ventilation as a potential contributor to patient-self inflicted lung injury in patients undergoing non-invasive ventilation (NIV) (18). Of note, accurate monitoring of airway pressure within NIV interfaces such as the helmet was demonstrated to be greatly valuable because the use of expiratory filters may hide some pressure within the hood—and then underestimate the airway pressure—despite a preset level of PEEP by using standard PEEP valves (19). This may allow us to titrate the NIV delivery pressures better.

## Oxygen saturation monitoring after tracheal extubation

Qiu et al. assessed the desaturation rate by using peripheral oxygen saturation monitoring in a randomized controlled study that compared the use of transnasal humidified rapid insufflation ventilator exchange (THRIVE group) immediately after extubation and the awake extubation (Control group). The THRIVE group decreased the incidence of desaturation of adverse hemodynamic events and increased patient comfort (Qiu et al.). This study confirmed the beneficial role of THRIVE reported in two randomized controlled trials conducted in patients undergoing non-laser laryngologic surgery (20) and in elderly patients undergoing general anesthesia (21).

## Author contributions

ER conceived and drafted the first version of the manuscript. LC and GB revised the manuscript. All authors contributed to the article and approved the submitted version.
